# Assessing the practice of data quality evaluation in a national clinical data research network through a systematic scoping review in the era of real-world data

**DOI:** 10.1093/jamia/ocaa245

**Published:** 2020-11-09

**Authors:** Jiang Bian, Tianchen Lyu, Alexander Loiacono, Tonatiuh Mendoza Viramontes, Gloria Lipori, Yi Guo, Yonghui Wu, Mattia Prosperi, Thomas J George, Christopher A Harle, Elizabeth A Shenkman, William Hogan

**Affiliations:** o1 Department of Health Outcomes and Biomedical Informatics, College of Medicine, University of Florida, Gainesville, Florida, USA; o2 Cancer Informatics Shared Resource, University of Florida Health Cancer Center, Gainesville, Florida, USA; o3 Clinical and Translational Institute, University of Florida, Gainesville, Florida, USA; o4 Department of Epidemiology, College of Public Health and Health Professions & College of Medicine, University of Florida, Gainesville, Florida, USA; o5 Hematology & Oncology, Department of Medicine, College of Medicine, University of Florida, Gainesville, Florida, USA

**Keywords:** data quality assessment, real-world data, clinical data research network, electronic health record, PCORnet

## Abstract

**Objective:**

To synthesize data quality (DQ) dimensions and assessment methods of real-world data, especially electronic health records, through a systematic scoping review and to assess the practice of DQ assessment in the national Patient-centered Clinical Research Network (PCORnet).

**Materials and Methods:**

We started with 3 widely cited DQ literature—2 reviews from Chan et al (2010) and Weiskopf et al (2013a) and 1 DQ framework from Kahn et al (2016)—and expanded our review systematically to cover relevant articles published up to February 2020. We extracted DQ dimensions and assessment methods from these studies, mapped their relationships, and organized a synthesized summarization of existing DQ dimensions and assessment methods. We reviewed the data checks employed by the PCORnet and mapped them to the synthesized DQ dimensions and methods.

**Results:**

We analyzed a total of 3 reviews, 20 DQ frameworks, and 226 DQ studies and extracted 14 DQ dimensions and 10 assessment methods. We found that completeness, concordance, and correctness/accuracy were commonly assessed. Element presence, validity check, and conformance were commonly used DQ assessment methods and were the main focuses of the PCORnet data checks.

**Discussion:**

Definitions of DQ dimensions and methods were not consistent in the literature, and the DQ assessment practice was not evenly distributed (eg, usability and ease-of-use were rarely discussed). Challenges in DQ assessments, given the complex and heterogeneous nature of real-world data, exist.

**Conclusion:**

The practice of DQ assessment is still limited in scope. Future work is warranted to generate understandable, executable, and reusable DQ measures.

## INTRODUCTION

There has been a surge of national and international clinical research networks (CRNs) curating immense collections of real-world data (RWD) from diverse sources of different data types such as electronic health records (EHRs) and administrative claims among many others. One prominent CRN example is the national Patient-Centered Clinical Research Network (PCORnet)^1^^,^^2^ funded by the Patient-Centered Outcomes Research Institute (PCORI) that contains more than 66 million patient data across the United States (US).^3^ The OneFlorida Clinical Research Consortium^4^ first created in 2009 is 1 of the 9 CRNs contributing to the national PCORnet. The OneFlorida network currently includes 12 healthcare organizations that provide care for more than 60% of Floridians through 4100 physicians, 914 clinical practices, and 22 hospitals covering all 67 Florida counties.^5^ The centerpiece of the OneFlorida network is its Data Trust, a centralized data repository that contains longitudinal and robust patient-level records of approximately15 million Floridians from various sources, including Medicaid and Medicare programs, cancer registries, vital statistics, and EHR systems from its clinical partners. Both the amount and types of data collected by OneFlorida is staggering.

Rising from the US Food and Drug Administration (FDA) Real-world Evidence (RWE) program, RWD such as those in the OneFlorida are increasingly important to support a wide range of healthcare and regulatory decisions.^6^^,^^7^ RWD are playing an increasingly critical role in various other national initiatives, such as the learning health systems,^8^^,^^9^ comparative effectiveness research,^10^ and programmatic clinical trials.^11^ Nevertheless, concerns over the quality of RWD, where data quality (DQ) issues, such as incompleteness, inconsistency, and accuracy, are widely reported and discussed.^12^^,^^13^ To maximize the utility of RWD, data quality should be systematically assessed and understood.

The literature on DQ assessment is rich with a number of DQ frameworks developed over time. Wang et al (1996)^14^ proposed a conceptual framework for assessing DQ aspects that are important to data consumers. McGilvray (2008)^15^ described 10 steps to quality data, where DQ assessment is an important step. Chan et al (2010)^16^ conducted a literature review on EHR DQ and summarized 3 DQ aspects: *accuracy*, *completeness,* and *comparability*. Nahm (2012)^17^ defined 10 DQ dimensions (eg, *accuracy*, *currency*, *completeness*) specific to clinical research with a framework for DQ practice. Kahn et al (2012)^18^ proposed the “*fit-for-use by data consumers*” concept with a process model for multisite DQ assessment. Weiskopf et al (2013a)^19^ provided an updated literature review on EHR DQ and identified 5 DQ dimensions: *completeness*, *correctness*, *concordance*, *plausibility,* and *currency*. They then focused on *completeness* in their follow up work (ie, Weiskopf et al [2013b]^20^). Liaw et al (2013)^21^ summarized the most reported dimensions in DQ assessment. Zozus et al (2014)^22^ conducted a literature review to identify DQ dimensions that affect the capacity of data to support research conclusions the most. Johnson et al (2015)^23^ developed an ontology to define DQ dimensions to enable automated computation of DQ measures. Garcí A-de-León-Chocano (2015)^24^ described a DQ assessment framework and constructed a set of processes. Kahn et al (2016)^25^ developed the “*harmonized data quality assessment terminology*” that organizes DQ assessment into 3 categories: *conformance*, *completeness,* and *plausibility*. Reimer et al (2016)^26^ developed a framework based on the 5 DQ dimensions from Weiskopf et al (2013a),^19^ with a focus on longitudinal data repositories. Khare et al (2017)^27^ summarized DQ issues and mapped to the harmonized DQ terms. Smith et al (2017)^28^ shared a framework for assessing the DQ of administrative data. Weiskopf et al (2017)^29^ developed a 3x3 DQ assessment guideline, where they selected 3 core dimensions from the 5 dimensions they defined in Weiskopf et al (2013a)^19^ and each dimension has 3 core DQ constructs. Lee et al (2018)^30^ modified the dimensions defined in Kahn et al (2016)^25^ to support specific research tasks. Feder (2018)^31^ described common DQ domains and approaches. Terry et al (2019)^32^ proposed a model for assessing EHR DQ, deriving from the 5 dimensions in Weiskopf et al (2013a).^19^ Nordo et al (2019)^33^ proposed outcome metrics in the use of EHR data, including measures related to DQ. Bloland et al (2019)^34^ offered a framework that describes immunization data in terms of 3 key characteristics (ie, data quality, usability, and utilization). Henley-Smith et al (2019)^35^ derived a 2-level DQ framework based on Kahn et al (2016).^25^ Charnock et al (2019)^36^ conducted a systematic review focusing on the importance of *accuracy* and *completeness* in secondary use of EHR data.

However, the literature on DQ assessment of EHR data is due for an update as the latest review article on this topic is from Weiskopf et al (2013a)^19^ that covered the literature before 2012. Further, few studies have assessed the practice of DQ assessment in large clinical networks. Callahan et al (2017)^37^ mapped the data checks in 6 clinical networks to their DQ assessment framework—the harmonized data quality assessment by Kahn et al (2016).^25^ One of the networks Callahan et al (2017)^37^ assessed is the Pediatric Learning Health System (PEDSnet), which also contributes to the national PCORNet like OneFlorida. Qualls et al (2018),^38^ from the PCORnet data coordinating center, presented the existing PCORnet DQ framework (ie, called “data characterization”), where they focused on only 3 DQ dimensions: data model conformance, data plausibility, and data completeness, initially with 13 DQ checks. They reported that the data characterization process they put in place has led to improvements in foundational DQ (eg, elimination of conformance errors, decrease in outliers, and more complete data for key analytic variables). As our OneFlorida network contributes to the PCORnet, we participate in the data characterization process. The data characterization process in PCORnet has evolved significantly since Qualls et al (2018).^38^ Thus, our study aims to identify gaps in the existing PCORnet data characterization process. To have a more complete picture of DQ dimensions and methods, we first conducted a systematic scoping review of existing DQ literature related to RWD. Through the scoping review, we organized the existing DQ dimensions as well as the methods used to assess these DQ dimensions. We then reviewed the DQ dimensions and corresponding DQ methods used in the PCORnet data characterization process (8 versions since 2016) to assess the DQ practice in PCORnet and how it has evolved.

## MATERIALS AND METHODS

We followed the typical systemic review process to synthesize relevant literature to extract DQ dimensions and DQ methods, mapped their relationships, and mapped them to the PCORnet data checks. Throughout the process, 2 team members (TL and AL) independently carried out the review, extraction, and mapping processes in each step, and disagreements between the 2 reviewers were first resolved through discussion with a third team member (JB) first and then the entire study team if necessary. We followed the Preferred Reporting Items for Systematic Reviews and Meta-Analyses (PRISMA) guideline and generated the PRIMSA flow diagram.

### A systematic scoping review of data quality assessment literature

We started with 3 widely cited core references on EHR DQ assessment, including 2 review articles from Chan et al (2010)^16^ and Weiskopf et al (2013a),^19^ and 1 DQ framework from Kahn et al (2016).^25^ First, we summarized and mapped the DQ dimensions in these 3 core references. We merged the dimensions that are similar in concept but named differently. For example, Chan et al (2010)^16^ defined “*data accuracy*” as whether the data “*can accurately reflect an underlying state of interest*,” while Weiskopf et al (2013a)^19^ defined it as “*data correctness*” (ie, “*whether the data is true*”). Then we synthesized the methods used to assess these DQ dimensions. Weiskopf et al (2013a)^19^ summarized the DQ assessment methods, while Chan et al (2010)^16^ and Kahn et al (2016)^25^ only provided definitions and examples on how to measure the different DQ dimensions. Thus, we mapped these definitions and examples to the methods reported in Weiskopf et al (2013a)^19^ according to their dimension definitions and measurement examples. For example, Chan et al (2010)^16^ defined “*completeness*” as “*the level of missing data*” and discussed various studies that have shown the variation in the amount of missing data across different data areas (eg, problem lists and medication lists) and clinical settings, while Kahn et al (2016)^25^ provided examples on how to measure “*completeness*” (eg, “*the encounter ID variable has missing values*”). Thus, we mapped “*completeness*” to the method of checking “*element presence*” (ie, “*whether or not desired data elements are present*”) defined in Weiskopf et al (2013a).^19^ We created new categories if the measurement examples cannot be mapped to existing methods in Weiskopf et al (2013a).^19^ For example, Kahn et al (2016)^25^ defined a “*conformance*” dimension that cannot be mapped to any of the methods defined in Weiskopf et al (2013a).^19^ Thus, we created a new method term “*conformance check*” to assess “*whether the values that are present meet syntactic or structural constraints.*” Kahn et al (2016)^25^ gave examples of *conformance check* such as the variable sex shall only have values: “Male,” “Female,” or “Unknown.”

We then reviewed the literature cited in the 3 core references. Chan et al (2010)^16^ and Weiskopf et al (2013a)^19^ reviewed individual papers that conducted DQ assessment experiments, while the DQ framework from Kahn et al (2016)^25^ is based on 9 other frameworks (however, full text of 1 framework is not available) and the literature review by Weiskopf et al (2013a).^19^ For completeness, we extracted the extra dimensions that were mentioned in the 8 frameworks but not included in the framework from Kahn et al (2016).^25^ We also summarized the methods for these additional dimensions according to the measurement examples given in the original frameworks.

We then reviewed the articles that were cited in the 2 core review papers: Chan et al (2010)^16^ and Weiskopf et al (2013a).^19^ We mapped the dimensions and methods mentioned in these articles to the ones we extracted from Kahn et al (2016).^25^ During this process, we revised the definitions of the dimensions and methods to make them more inclusive of the different literature.

Weiskopf et al (2013a)^19^ is the latest review article that covers DQ literature before January 2012. Thus, we conducted an additional review of DQ assessment literature published after 2012 to February 2020. We identified 2 group of search keywords (ie, DQ-related and EHR-related keywords) mainly from the 3 core references. The search strategy including the keywords is detailed in the Supplementary Appendix A. An article was included if it assessed the quality of data derived from EHR systems using clearly defined DQ measurements (even if the primary goal of the study was not to assess DQ).

We then extracted the DQ dimensions and methods from these new articles, merged the ones that are similar to the existing ones, and created new dimensions and methods if necessary. After this process, we created a comprehensive list of dimensions, their concise definitions, and the methods commonly used to assess these DQ dimensions.

### Map the PCORnet data characterization checks to the data quality dimensions and methods

We reviewed the measurements in the PCORnet data checks (from version 1 published in 2016 to version 8 as of 2020)^38^^,^^39^ and mapped them to the dimensions and methods we summarized above. Two reviewers (TL and AL) independently carried out the mapping tasks, and conflicts were resolved by a third reviewer (JB) through group discussions.

## RESULTS

### Data quality dimensions and assessment methods summarized from the 3 core references

#### Data quality dimensions

Overall, we extracted 12 dimensions (ie, *currency*, *correctness/accuracy*, *plausibility*, *completeness*, *concordance*, *comparability*, *conformance*, *flexibility*, *relevance*, *usability/ease-of-use*, *security*, and *information loss and degradation*) from the 3 core references and then mapped the relationships among them.

Chan et al (2010)^16^ conducted a systematic review on EHR DQ literature from January 2004 to June 2009 focusing on how DQ affects quality of care measures. They extracted 3 DQ aspects: (1) *accuracy*, including data currency and granularity; (2) *completeness*; and (3) *comparability*.

Weiskopf et al (2013a)^19^ performed a literature review of EHR DQ assessment methodology, covering articles published before February 2012. They identified 27 unique DQ terms/dimensions. After merging DQ terms with similar definitions and excluding dimensions that have no measurement (ie, how the DQ dimension is measured), they retained 5 dimensions: (1) *completeness*, (2) *correctness*, (3) *concordance*, (4) *plausibility*, and (5) *currency*.

Kahn et al (2016)^25^ proposed a DQ assessment framework for secondary use of EHR data, consisting of 3 DQ dimensions: (1) *conformance* with 3 subcategories: *value conformance*, *relational conformance*, and *computational conformance*; (2) *completeness*; and (3) *plausibility* with 3 subcategories: *uniqueness plausibility*, *atemporal plausibility*, and *temporal plausibility*. Each DQ dimension can be assessed in 2 different DQ assessment contexts: *verification* (ie, “*how data values match expectations with respect to metadata constraints, system assumptions, and local knowledge*”), and *validation* (ie, “*the alignment of data values with respect to relevant external benchmarks*”).

For comprehensiveness, we also reviewed the 8 DQ frameworks that were cited by Kahn et al (2016)^25^ and included any DQ new dimension that has been reported in at least 2 of the 8 DQ frameworks. A total of 5 additional dimensions was identified: (1) *flexibility* from Wang et al (1996);^14^ (2) *relevance* from Liaw et al (2013);^21^ (3) *usability/ease-of-use* from McGilvray (2008);^15^ (4) *security* from Liaw et al (2013);^21^ and (5) *information loss and degradation* from Zozus et al (2014).^22^

#### Data quality assessment methods

A total of 10 DQ assessment methods were identified: 7 from Weiskopf et al (2013a),^19^ 1 from Chan et al (2010)^16^ and Kahn et al (2016),^25^ and 2 from the 8 frameworks referred by Kahn et al (2016).^25^

Out of the 3 core references, only Weiskopf et al (2013a)^19^ explicitly summarized 7 DQ assessment methods, including (1) *gold standard*; (2) *data element agreement*; (3) *element presence*; (4) *data source agreement*; (5) *distribution comparison*; (6) *validity check*; and (7) *log review*.

From the other 2 core references, we summarized 3 new DQ assessment methods: (1) *conformance check* from both Chan et al (2010)^16^ and Kahn et al (2016);^25^ (2) *qualitative assessment* from Liaw et al (2013)^21^ a DQ framework referenced in Kahn et al (2016);^25^ and (3) *security analysis* from Liaw et al (2013).^21^

### Review of individual data quality assessment studies with updated literature search

**Figure 1.**
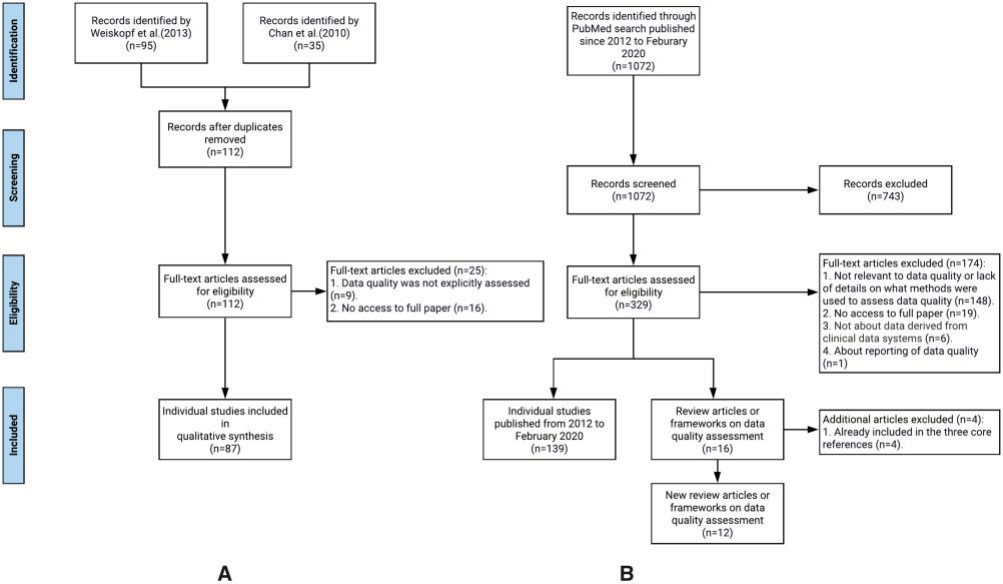
The flow chart of the literature review process: (A) individual studies identified from Chan et al (2010) and Weiskopf et al (2013a), and (B) new data quality related articles (both individual studies and review/framework articles) published from 2012 to February 2020.

We first reviewed 87 individual DQ assessment studies cited in the 2 systematic review articles: Chan et al (2010)^16^ and Weiskopf et al (2013a),^19^ extracted the DQ measurements used and mapped them to the 12 DQ dimensions and 10 DQ assessment methods. Through this process, we revised the definitions of the DQ dimensions and methods if necessary. Figure 1A shows our review process.

Further, since the review from Weiskopf et al (2013a)^19^ only covered the literature before 2012, we conducted an additional review of the literature on EHR DQ assessment published from 2012 up until February 2020. Figure 1B illustrates our literature search process following the PRISMA flow diagram.

Through this process, we identified 1072 publications and then excluded 743 articles through title and abstract screening. During the full-text screening, 172 articles were excluded because either (1) the full text was not accessible (n = 19); (2) the paper was not relevant to DQ, or the paper lacks sufficient details on what methods were used to assess DQ (n = 147); or (3) the data of interest were not derived from clinical data systems (n = 6). At the end, 157 new articles were included, out of which 139 were individual studies and 16 were review articles or frameworks. Four of the 16 review/framework articles were already included the 3 core references, thus, effectively, we identified 12 new review or framework articles. We effectively reviewed 139 new individual DQ assessment studies published after 2012 until February 2020. The list of all reviewed articles is in Supplementary Appendix B.

#### Review of the newly identified DQ frameworks and review articles

From the 12 newly identified DQ frameworks or reviews, we extracted the DQ dimensions and assessment methods and mapped them to the existing 12 DQ dimensions and 10 methods we extracted from the 3 core references. We refined the original definitions if necessary. We did not identify any new DQ methods, but we identified 2 new DQ dimensions: (1) *consistency* (ie, “*pertains to the constancy of the data, at the desired degree of detail for the study purpose, within and across databases and data sets*” from Feder [2018]^31^) and (2) *understandability/interpretability* (ie, “*the ease with which a user can understand the data*” from Smith et al [2017]^28^) The concept of *consistency* from Feder (2018)^31^ can be connected to *concordance* in Weiskopf et al (2013a)^19^ and various other dimensions (eg, *plausibility* from Kahn et al [2016]^25^) especially *comparability* from Chan et al (2010).^16^ Nevertheless, *consistency* based on the definitions and examples from Feder (2018)^31^ covers a broader and more abstract concept pertaining to the constancy (ie, “*the quality of being faithful and dependable*”) of the data.

#### Review of individual studies published after 2012

**Figure 2.**
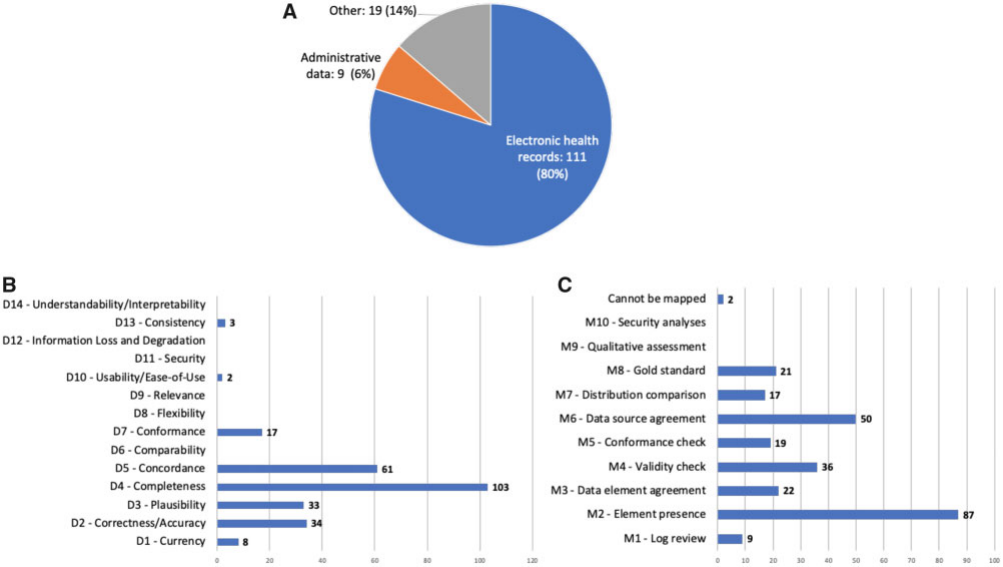
The numbers of studies by (A) data type, (B) DQ dimension, and (C) DQ assessment method.

For the 139 individual studies, we extracted the type of the data (eg, EHR or claims), the DQ dimensions, and assessment methods including the specific DQ measurements if mentioned. Figure 2 shows the results. No new DQ dimension and assessment methods were identified from these studies.

### A summary of DQ dimensions and assessment methods

**Figure 3.**
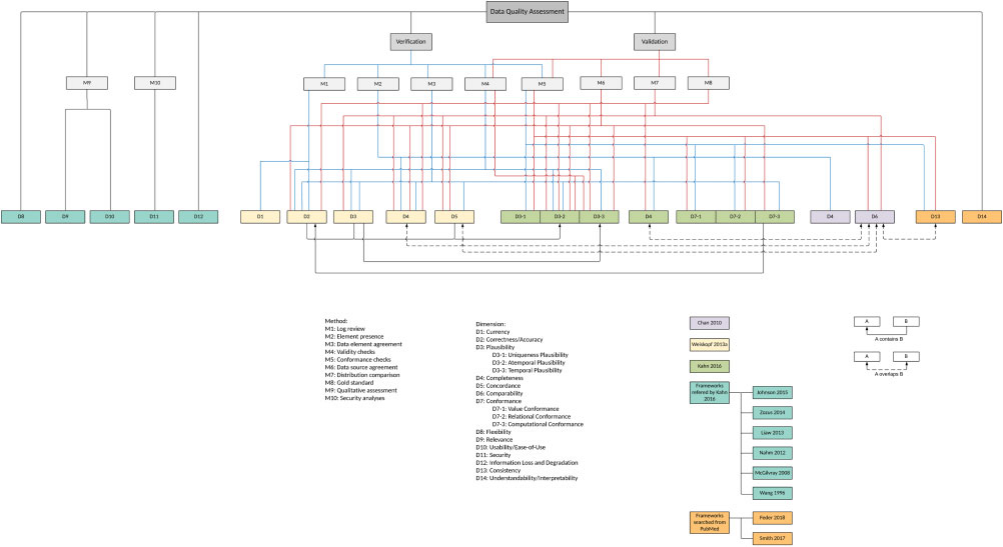
A summarization of existing DQ dimensions and DQ assessment methods.

We summarized the 14 DQ dimensions and 10 DQ assessment methods and mapped the relationships among them as shown in Figure 3. Following Kahn et al (2016),^25^ we categorized the DQ dimensions and methods into 2 contexts: verification (ie, can be assessed using the information within the dataset or using common knowledge) and validation (ie, can be assessed using external resources such as compared with external data sources and checked against data standards). However, 6 DQ dimensions (ie, flexibility, relevance, usability, security, information loss and degradation, and understandability/interpretability) and 2 DQ assessment methods (ie, qualitative assessment and security analyses) cannot be categorized into either context.

In the broader DQ literature, there is also the concept of intrinsic DQ versus extrinsic DQ.^14^^,^^40^ The intrinsic DQ denotes that “*data have quality in its own right*”^14^ and “*independent of the context in which data is produced and used*,”^40^ while the extrinsic DQ, although not explicitly defined, are more sensitive to the external environments, considering the context of the task at hand (ie, contextual DQ^40^) and the information systems that store and deliver the data (ie, accessibility DQ and representational DQ^40^) In our context, D1—D7 are more related to intrinsic DQ; while D8—D14 may fall into the extrinsic DQ category. Note that there is also literature that defines intrinsic DQ versus extrinsic DQ in terms of how they can be assessed (ie, “*this measure is called intrinsic if it does not require any additional data besides the dataset, otherwise it is called extrinsic*”^41^); however, such definitions may be incomplete and imprecise. For example, *correctness/accuracy* (D2) is part of the intrinsic DQ defined in Strong et al (1997)^40^ but can be assessed with external datasets in the context of validation.

Tables 1 and 2 show the definitions and the reference frameworks or reviews from which we extracted the definitions for DQ dimensions and DQ methods, respectively.

**Table 1:** Data quality dimensions summarized from existing DQ frameworks and reviews

	Dimension	Definition	Source frameworks/reviews
D1	Currency	Data were considered current if they were recorded in the EHR within a reasonable period of time following measurement or, alternatively, if they were representative of the patient state at a desired time of interest. Weiskopf et al (2013a)^19^	Bloland et al (2019),^34^ Nordo et al (2019),^33^ Terry et al (2019),^32^ Feder SL (2018),^31^ Smith et al (2017),^28^ Weiskopf et al (2017),^29^ Johnson et al (2015),^23^ Liaw et al (2013),^21^ Weiskopf et al (2013a),^19^ Nahm (2012),^17^ McGilvray (2008),^15^ Wang et al (1996)^14^
D2	Correctness/Accuracy	EHR data were considered correct when the information they contained was true. Weiskopf et al (2013a)^19^	Bloland et al (2019),^34^ Nordo et al (2019),^33^ Terry et al (2019),^32^ Feder SL (2018),^31^ Weiskopf et al (2017),^29^ Smith et al (2017),^28^ Garcí A-de-León-Chocano (2015),^24^ Johnson et al (2015),^23^ Zozus et al (2014),^22^ Liaw et al (2013),^21^ Weiskopf et al (2013a),^19^ Nahm (2012),^17^ Chan et al (2010),^16^ McGilvray (2008),^15^ Wang et al (1996)^14^
D3	Plausibility	Plausibility focuses on actual values as a representation of a real-world object or conceptual construct by examining the distribution and density of values or by comparing multiple values that have an expected relationship to each other. Kahn et al (2016)^25^	Henley-Smith et al (2019),^35^ Bloland et al (2019),^34^ Feder SL (2018),^31^ Lee et al (2018),^30^ Khare et al (2017),^27^ Kahn et al (2016),^25^ Weiskopf et al (2013a)^19^
D3-1*	Uniqueness Plausibility	The Uniqueness subcategory seeks to determine if objects (entities, observations, facts) appear multiple times in settings where they should not be duplicated or cannot be distinguished within a database (Verification) or when compared with an external reference (Validation). Kahn et al (2016)^25^	Henley-Smith et al (2019),^35^ Lee et al (2018),^30^ Kahn et al (2016),^25^ Garcí A-de-León-Chocano (2015),^24^ Zozus et al (2014),^22^ McGilvray (2008)^15^
D3-2*	Atemporal Plausibility	Atemporal Plausibility seeks to determine if observed data values, distributions, or densities agree with local or “common” knowledge (Verification) or from comparisons with external sources that are deemed to be trusted or relative gold standards (Validation). Kahn et al (2016)^25^	Henley-Smith et al (2019),^35^ Lee et al (2018),^30^ Smith et al (2017),^28^ Kahn et al (2016)^25^, Johnson et al (2015),^23^ Zozus et al (2014),^22^ Nahm (2012),^17^ McGilvray (2008)^15^
D3-3*	Temporal Plausibility	Temporal plausibility seeks to determine if time-varying variables change values as expected based on known temporal properties or across 1 or more external comparators or gold standards. Kahn et al (2016)^25^	Henley-Smith et al (2019),^35^ Lee et al (2018),^30^ Smith et al (2017),^28^ Kahn et al (2016)^25^
D4	Completeness	Completeness focuses on features that describe the frequencies of data attributes present in a data set without reference to data values. Kahn et al (2016)^25^	Henley-Smith et al (2019),^35^ Bloland et al (2019),^34^ Nordo et al (2019),^33^ Terry et al (2019),^32^ Feder SL (2018),^31^ Lee et al (2018),^30^ Weiskopf et al (2017),^29^ Smith et al (2017),^28^ Khare et al (2017),^27^ Reimer et al (2016),^26^ Kahn et al (2016),^25^ Garcí A-de-León-Chocano (2015),^24^ Johnson et al (2015),^23^ Zozus et al (2014),^22^ Weiskopf et al (2013b),^20^ Weiskopf et al (2013a),^19^ Kahn et al (2012),^18^ Nahm (2012),^17^ Chan et al (2010),^16^ McGilvray (2008),^15^ Wang et al (1996)^14^
D5	Concordance	Is there agreement between elements in the EHR, or between the EHR and another data source? Weiskopf et al (2013a)^19^	Bloland et al (2019),^34^ Smith et al (2017),^28^ Reimer et al (2016),^26^ Weiskopf et al (2013a)^19^
D6	Comparability	Comparability is similarity in data quality and availability for specific data elements used in a measure across different entities, such as health plans or physicians or data sources. Chan et al (2010)^16^	Terry et al (2019),^32^ Chan et al (2010)^16^
D7	Conformance	Whether the values that are present meet syntactic or structural constraints. Kahn et al (2016)^25^	Henley-Smith et al (2019),^35^ Lee et al (2018),^30^ Khare et al (2017),^27^ Kahn et al (2016)^25^
D7-1*	Value Conformance	Agreement with a prespecified, constraint-driven data architecture. Kahn et al (2016)^25^	Henley-Smith et al (2019),^35^ Nordo et al ^(^2019),^33^ Lee et al (2018),^30^ Smith et al (2017),^28^ Kahn et al (2016),^25^ Garcí A-de-León-Chocano (2015),^24^ Johnson et al (2015),^23^ Nahm (2012),^17^ Wang et al (1996)^14^
D7-2*	Relational Conformance	Agreement with additional structural constraints imposed by the physical database structures that store data values. Kahn et al (2016)^25^	Henley-Smith et al (2019),^35^ Lee et al (2018),^30^ Kahn et al (2016),^25^ Garcí A-de-León-Chocano (2015),^24^ Johnson et al (2015),^23^ Zozus et al (2014),^22^ Nahm (2012),^17^ McGilvray (2008)^15^
D7-3*	Computational Conformance	If computations used to create derived values from existing variables yield the intended results either within a data set (Verification) or between data sets (Validation), when programs are based on identical specifications. Kahn et al (2016)^25^	Henley-Smith et al (2019),^35^ Lee et al (2018),^30^ Kahn et al (2016)^25^
D8	Flexibility	The extent to which data are expandable, adaptable, and easily applied to many tasks. Wang et al (1996)^14^	Johnson et al (2015),^23^ Wang et al (1996)^14^
D9	Relevance	The extent to which information is applicable and helpful for the task at hand. Liaw et al (2013)^21^	Bloland et al (2019),^34^ Johnson et al (2015),^23^ Liaw et al (2013),^21^ Nahm (2012),^17^ McGilvray (2008),^15^ Wang et al (1996)^14^
D10	Usability/Ease-of-Use	A measure of the degree to which data can be accessed and used and the degree to which data can be updated, maintained, and managed. McGilvray (2008)^15^	Liaw et al (2013)^21^, McGilvray (2008),^15^ Wang et al (1996)^14^
D11	Security	Personal data is not corrupted, and access suitably controlled to ensure privacy and confidentiality. Liaw et al (2013)^21^	Liaw et al (2013),^21^ Wang et al (1996)^14^
D12	Information Loss and Degradation	The loss and degradation of information content over time. Zozus et al (2014)^22^	Bloland et al (2019),^34^ Zozus et al (2014),^22^ McGilvray (2008)^15^
D13	Consistency	Pertains to the constancy of the data, at the desired degree of detail for the study purpose, within and across databases and data sets. Feder SL (2018)^31^	Feder SL (2018),^31^ Smith et al (2017)^28^
D14	Understandability/Interpretability	The ease with which a user can understand the data. Smith et al (2017)^28^	Smith et al (2017),^28^ Wang et al (1996)^14^

* D3-1, D3-2, and D3-3 are subcategories of D3; D7-1, D7-2, and D7-3 are subcategories of D7.

**Table 2.** Data quality assessment methods summarized from existing DQ frameworks and reviews

	Method	Definition	Source frameworks/reviews
M1	Log review	Information on the actual data entry practices (eg, dates, times, edits) is examined. Weiskopf et al (2013a)^19^	Bloland et al (2019),^34^ Feder SL (2018),^31^ Weiskopf et al (2017),^29^ Liaw et al (2013),^21^ Weiskopf et al (2013a),^19^ Nahm (2012)^17^
M2	Element presence	A determination is made as to whether or not desired or expected data elements are present. Weiskopf et al (2013a)^19^	Henley-Smith et al (2019),^35^ Bloland et al (2019),^34^ Terry et al (2019),^32^ Lee et al (2018),^30^ Weiskopf et al (2017),^29^ Khare et al (2017),^27^ Reimer et al (2016),^26^ Kahn et al (2016),^25^ Johnson et al (2015),^23^ Liaw et al (2013),^21^ Weiskopf et al (2013a),^19^ Nahm (2012),^17^ Chan et al (2010)^16^
M3	Data element agreement	Two or more elements within an EHR are compared to see if they report the same or compatible information. Weiskopf et al, (2013a)^19^; derived (calculated) values from existing variables yield the intended results within a data set. Kahn et al (2016)^25^	Henley-Smith et al (2019),^35^ Bloland et al (2019),^34^ Feder SL (2018),^31^Lee et al (2018),^30^ Weiskopf et al (2017),^29^ Reimer et al (2016),^26^ Kahn et al (2016),^25^ Nahm (2012)^17^
M4	Validity check	If observed data values or densities agree with “common” knowledge or external knowledge; if time varying variables change values as expected based on known temporal properties or external knowledge. Kahn et al (2016)^25^	Henley-Smith et al (2019),^35^ Bloland et al (2019),^34^ Terry et al (2019),^32^ Feder SL (2018),^31^ Lee et al (2018),^30^ Weiskopf et al (2017),^29^ Khare et al (2017),^27^ Kahn et al (2016),^25^ Weiskopf et al (2013a)^19^
M5	Conformance check	Check the uniqueness of objects which should not be duplicated; the dataset agreement with prespecified or additional structural constraints Kahn et al (2016);^25^ and the agreement of object concepts and formats granularity) between 2 or more data sources.	Henley-Smith et al (2019),^35^ Feder SL (2018),^31^ Lee et al (2018),^30^ Khare et al (2017),^27^ Kahn et al (2016),^25^ Johnson et al (2015),^23^ Liaw et al (2013),^21^ Weiskopf et al (2013a),^19^ Nahm (2012),^17^ Chan et al (2010)^16^
M6	Data source agreement	Data from the EHR are compared with data from another source to determine if they are in agreement Weiskopf et al (2013a);^19^ derived (calculated) values from existing variables yield the intended results or between data sets when programs are based on identical specifications. Kahn et al (2016)^25^	Bloland et al (2019),^34^ Terry et al (2019),^32^ Feder SL (2018),^31^ Reimer et al (2016)^26^
M7	Distribution comparison	Distributions or summary statistics of aggregated data from the EHR are compared with the expected distributions for the clinical concepts of interest. Weiskopf et al (2013a)^19^	Terry et al (2019),^32^ Feder SL (2018),^31^ Weiskopf et al (2017),^29^ Kahn et al (2016),^25^ Liaw et al (2013),^21^ Weiskopf et al (2013a),^19^ Chan et al (2010)^16^
M8	Gold standard	Data value and presence in the dataset is the same as the value and presence from trusted reference standards or datasets. If the data is extracted from paper record in a rigorous fashion, then it’s a gold standard (eg, manual chart review).	Bloland et al (2019),^34^ Terry et al (2019),^32^ Feder SL (2018),^31^ Kahn et al (2016),^25^ Weiskopf et al (2013a),^19^ Nahm (2012)^17^
M9	Qualitative assessment	Descriptive qualitative measures with group interviews and interpreted with grounded theory. Liaw et al (2013)^21^	Liaw et al (2013)^21^
M10	Security analyses	Analyses of access reports to examine whether there’s security issue. Liaw et al (2013)^21^	Liaw et al (2013)^21^

### Map the PCORnet data characterization checks to the synthesized DQ dimensions and methods

Table 3 shows the result of mapping existing PCORnet data characterization checks to the 14 DQ dimensions and 10 DQ assessment methods.

**Table 3.** Mapping PCORnet data characterization checks to the 14 DQ dimensions and 10 DQ assessment methods

Data Check (DC)	Working description^a^	Status	Method	Dimension
DC 1.01	Required tables are not present	Since version 1	M2	D4, D7
DC 1.02	Required tables are not populated	Since version 1	M2	D4, D7
DC 1.03	Required fields are not present	Since version 1	M2	D4, D7
DC 1.04	Required fields do not conform to data model specifications for data type, length, or name.	Since version 1	M5	D7-1, D7-2
DC 1.05	Tables have primary key definition errors	Since version 1	M5	D3-1, D7-2
DC 1.06	Required fields contain values outside of specifications	Since version 1	M5	D7-1
DC 1.07	Required fields have non-permissible missing values	Since version 1	M2	D4
DC 1.08	Tables contain orphan PATIDs	Added in version 2	M2, M5	D4, D5, D7-2
DC 1.09	Tables contain orphan ENCOUNTERIDs	Added in version 2	M2, M5	D4, D5, D7-2
DC 1.10	Replication errors between the ENCOUNTER, PROCEDURES and DIAGNOSIS tables	Added in version 2	M5	D3-1, D7-2
DC 1.11	> 5% of encounters are assigned to more than 1 patient	Added in version 3	M5	D3-1, D7-2
DC 1.12	Tables contain orphan PROVIDERIDs	Added in version 5	M2, M5	D4, D5, D7-2
DC 1.13	More than 5% of ICD, CPT, LOINC, RXCUI, or NDC codes do not conform to the expected length or content	Added in version 6	M5	D7-1, D7-2
DC 1.14	Patients in the DEMOGRAPHIC table are not in the HASH_TOKEN table	Added in version 8	M2, M5	D4, D5, D7-2
DC 2.01	More than 5% of records have future dates	Since version 1	M4	D2, D3-3
DC 2.02	> 10% of records fall into the lowest or highest categories of age, height, weight, diastolic blood pressure, systolic blood pressure, or dispensed days supply	Since version 1	M4, M7	D3-2
DC 2.03	More than 5% of patients have illogical date relationships	Added in version 2	M4	D2, D3-3
DC 2.04	The average number of encounters per visit is > 2.0 for inpatient (IP), emergency department (ED), or ED to inpatient (EI) encounters	Added in version 2	M4, M7	D3-2
DC 2.05	More than 5% of results for selected laboratory tests do not have the appropriate specimen source	Added in version 3	M4, M5	D4, D7
DC 2.06	The median lab result value for selected tests is an outlier.	Added in version 5	M4	D3-2
DC 2.07	The average number of principal diagnoses per encounter is above threshold (2.0 for inpatient [IP] and ED to inpatient [EI])	Added in version 5	M4, M7	D3-2
DC 2.08	The monthly volume of encounter, diagnosis, procedure, vital, prescribing, or laboratory records is an outlier.	Added in version 7	M4, M7	D3-2
DC 3.01	The average number of diagnoses records with known diagnosis types per encounter is below threshold (1.0 for ambulatory [AV], inpatient [IP], emergency department [ED], or ED to inpatient [EI] encounters)	Since version 1	M4, M7	D3-2
DC 3.02	The average number of procedure records with known procedure types per encounter is below threshold (0.75 for ambulatory [AV] encounters, 0.75 for emergency department [ED] encounters, 1.00 for ED to inpatient [EI] encounters, and 1.00 for inpatient [IP] encounters]	Since version 1	M4, M7	D3-2
DC 3.03	More than 10% of records have missing or unknown values for the following fields: BIRTH_DATE, SEX, DISCHARGE_DISPOSITION, among others	Since version 1	M2	D4
DC 3.04	Less than 50% of patients with encounters have DIAGNOSIS records	Added in version 2	M2	D4
DC 3.05	Less than 50% of patients with encounters have PROCEDURES records	Added in version 2	M2	D4
DC 3.06	More than 10% of IP (inpatient) or ED to inpatient (EI) encounters with any diagnosis don't have a principal diagnosis	Added in version 2	M2	D4
DC 3.07	Encounters, diagnoses, or procedures in an ambulatory (AV), emergency department (ED), ED to inpatient (EI), or inpatient (IP) setting are less than 75% complete 3 months prior to the current month	Added in version 3	M2	D1, D4
DC 3.08	Less than 80% of prescribing orders are mapped to a RXNORM_CUI which fully specifies the ingredient, strength and dose form	Added in version 3	M2	D4
DC 3.09	Less than 80% of laboratory results are mapped to LAB_LOINC	Added in version 3	M2	D4
DC 3.10	Less than 80% of quantitative results for tests mapped to LAB_LOINC fully specify the normal range	Added in version 3	M2	D4
DC 3.11	Vital, prescribing, or laboratory records are less than 75% complete 3 months prior to the current month	Added in version 4	M2	D1, D4
DC 3.12	Less than 80% of quantitative results for tests mapped to LAB_LOINC fully specify the RESULT_UNIT	Added in version 5	M2	D4
DC 3.13	The percentage of patients with selected lab tests is below threshold	Added in version 8	M4, M7	D3-2, D4
DC 4.01	More than a 5% decrease in the number of patients or records in a CDM table	Added in version 6	M2	D12
DC 4.02	More than a 5% decrease in the number of patients or records for diagnosis, procedures, labs or prescriptions during an ambulatory (AV), other ambulatory (OA), emergency department (ED), or inpatient (IP) encounter	Added in version 6	M2	D12
DC 4.03	More than a 5% decrease in the number of records or distinct codes for ICD9 or ICD10 diagnosis or procedure codes or CPT/HCPCS procedure codes	Added in version 6	M2	D12
DC 4.01	DataMart's DIAGNOSIS table has a minimum ADMIT_DATE after January 2010. DataMarts should include data that can be well curated. When possible, DataMarts should include historical data from no later than 2010 to the present.	Since version 1, but removed in version 2	M2	D1
DC 4.02	DataMart's PROCEDURES table has a minimum ADMIT_DATE after January 2010. DataMarts should include data that can be well curated. When possible, DataMarts should include historical data from no later than 2010 to the present.	Since version 1, but removed in version 2	M2	D1
DC 4.03	DataMart's VITAL table has a minimum MEASURE_DATE after January 2010. DataMarts should include data that can be well curated. When possible, DataMarts should include historical data from no later than 2010 to the present.	Since version 1, but removed in version 2	M2	D1
DC 4.04	DataMart does not include all of the following encounter types: ambulatory (AV), inpatient (IP or EI), and emergency department (ED or EI) encounters. This complement of encounter types is not required but may be important for some research studies.	Since version 1, but removed in version 2	M2	D4
DC 4.05	DataMart has obfuscated or imputed	Since version 1, but removed in version 2	M10	D11

a Data in the PCORnet follows the PCORnet common data model (CDM). Both the PCORnet CDM and the PCORnet data checks specifications are available at https://pcornet.org/data-driven-common-model/.

## DISCUSSION

Evident from the large number of studies we identified—3 review articles, 20 DQ frameworks, and 226 DQ relevant studies—the literature on the quality of real-world clinical data, such as EHR and claims, for secondary research use is rich. Nevertheless, the definitions of and the relationships among the different DQ dimensions are not as clear as they could have been. For example, even though we merged *accuracy* with *correctness* into 1 DQ dimension as *accuracy/correctness* (D2), the original *accuracy* dimension (ie, “*the extent to which data accurately reflects an underlying state of interest includes timeliness and granularity*”) as defined by Chan et al (2010)^16^) actually contains both *correctness* (ie, “*data were considered correct when the information they contained was true*”) and *plausibility* (ie, “*actual values as a representation of a real-world*”) defined by Weiskopf et al (2013a)^19^ and Kahn et al (2016),^25^ respectively. Further, some DQ dimensions are quite broad and have overlapping concepts with other dimensions. For example, *comparability* can be mapped to *completeness*, *concordance*, *and consistency* depending on the perspectives (eg, frequency or value of a data element).

In terms of DQ assessment methods, similar overlapping definitions exist. For example, the difference between the concept of *distribution comparison* (M7) and *validity check* (M4) is subtle, where the original definition of *distribution comparison* in Weiskopf et al (2013a)^19^ refers to comparing a data element to an external authoritative resource (eg, comparing the prevalence of diabetes patients calculated from an EHR system to the general diabetes prevalence of that area), while *validity check* defined in Kahn et al (2016)^25^ refers to whether the value of a data element is out of the normal range (ie, outliers).

The practice of DQ assessment is not evenly distributed. As shown in Figure 2, most studies that mentioned DQ assessments focused on *completeness* (D4), *concordance* (D5), *correctness/accuracy* (D2), and *plausibility* (D3); while the *element presence* (M2), *data source agreement* (M6), *validity check* (M4), and *data element agreement* (M3) are the most used DQ methods, reflecting what aspects of DQ are important in real-world studies. We have similar observations examining the DQ assessment practice in the PCORnet. As shown in Tables 3 and 4, out of all the data checks in the PCORnet data characterization process, the most used data checks are *element presence* (M2, 25 checks), *validity check* (M4, 11 checks), and *conformance check* (M5, 11 checks), and the most examined DQ dimensions are *completeness* (D4, 21 checks), *conformance* (D7, 16 checks), and *plausibility* (D3, 13 checks), which raises the question why other DQ dimensions and DQ methods are not widely used in practice, especially in a CRN environment.

**Table 4.** The numbers of PCORnet data checks mapped to each DQ dimension and DQ assessment method

DQ assessment method	Number of DCs	DQ dimension	Number of DCs
M1 Log review	1	D1 Currency	9
M2 Element presence	25	D2 Correctness/Accuracy	2
M3 Data element agreement	0	D3 Plausibility	13
M4 Validity check	11	D4 Completeness	21
M5 Conformance check	11	D5 Concordance	4
M6 Data source agreement	0	D6 Comparability	0
M7 Distribution comparison	7	D7 Conformance	14
M8 Gold standard	0	D8 Flexibility	0
M9 Qualitative assessment	0	D9 Relevance	0
M10 Security analyses	1	D10 Usability/Ease-of-Use	0
		D11 Security	1
		D12 Information Loss and Degradation	3
		D13 Consistency	0
		D14 Understandability/Interpretability	0

Abbreviations: DC, data check; DQ, data quality.

The reason maybe multifold. First, the data from different sites of a CRN are heterogeneous in syntax (eg, file formats), schema (eg, data models and structures), and even semantics (eg, meanings or interpretations of the variables). This is not only because of the difference between different EHR vendors (eg, Cerner vs Epic), but also the difference in the implementation of the same EHR vendor system. For example, Epic’s flexibility in being able to create arbitrary flow sheets to meet different use cases also created inconsistency in data capturing at the data sources. Common data models (CDMs) and common data elements are common approaches to address these inconsistencies through transforming the source data into an interoperability common data framework. However, it is worth noting that standardization and harmonization of heterogeneous data sources are always difficult after the fact, when the data have already been collected. For example, in the OneFlorida network, although partners are required to provide a data dictionary of their source data, the units of measures are often neglected by the partners, leading to situations such as the average heights of patients being vastly higher than conventional wisdom. Our investigation of this DQ issue revealed that certain partners used centimeters rather than inches (as dictated by the PCORnet CDM) as the unit of measure. These “human” errors are inevitable, where a rigorous DQ assessment process is critical to identify these issues. Second, even though DQ is widely recognized as an important aspect, it is difficult to have a comprehensive process to capture all DQ issues from the get-go. The approach that the PCORnet takes is to have different levels of DQ assessment processes, where the general data checks (as shown in Table 3) are used to capture common and easy-to-catch errors while a study-specific data characterization process is used to inform whether the data at hand can inform a study’s specific objectives. Third, some DQ dimensions and DQ methods, although easy to understand in concept, are difficult to put in place and execute in reality. For example, *usability/ease-of-use* (D10) and *security* (D11), although straightforward to understand, lack well-defined executable measures. However, these DQ dimensions are still important aspects of DQ, and more efforts on methods and tools to assess DQ dimensions, such as *flexibility* (D8), *usability/ease-of-use* (D10), *security* (D11), and *understandability/interpretability* (D14), are needed to fill these knowledge gaps.

There are also a few studies^21^^,^^23^ that attempted to develop ontologies of DQ to “*enable automated computation of data quality measures*” and to “*make data validation more common and reproducible.*” However these efforts, although much needed, have not led to wide adoption. The “*harmonized data quality assessment terminology*” proposed by Kahn et al (2016),^25^ although not comprehensive, covers common and important aspects that matter in DQ assessment practice. Further expansion is warranted. Another interesting observation is that out of the 226 DQ assessment studies, only 1 study^42^ discussed the importance of reporting DQ assessment reports. It recommends, and we agree, that “*reporting on both general and analysis-specific data quality features*” are critical to ensure transparency and consistency in computing, reporting, and comparing DQ of different datasets. These aspects of DQ assessment also deserve further investigations.

## LIMITATIONS

First, we only used PubMed to search for relevant articles, thus, we may have missed some potentially relevant studies indexed in other databases (eg, Web of Science). Second, our review focused on qualitatively synthesizing DQ dimensions and DQ assessment methods but did not go into the details about how these DQ dimensions and methods can be applied. Further comprehensive investigation on which DQ checks and measures are concrete and executable is also warranted.

## CONCLUSIONS

Our review highlights the wide awareness and recognition of DQ issues in RWD, especially EHR data. Although the practice of DQ assessment in exists, it is still limited in scope. With the rapid adoption and increasing promotion of research using RWD, DQ issues will be increasingly important and call for attention from the research communities. However, different strategies of DQ may be needed given the complex and heterogeneous nature of RWD. DQ issues should not be treated alone but rather in full consideration with other data-related issues, such as selection bias among others. The addition of reporting DQ into the now widely recognized FAIR (ie, Findability, Accessibility, Interoperability, and Reuse) data principles may benefit the broader research community. Nevertheless, future work is warranted to generate understandable, executable, and reusable DQ measures and their associated assessments.

## FUNDING

This work was mainly supported by the University of Florida’s Creating the Healthiest Generation—Moonshot initiative and also supported in part by National Institutes of Health (NIH) grants UL1TR001427, R01CA246418, R21AG068717, as well as Patient-Centered Outcomes Research Institute (PCORI) grants PCORI ME-2018C3-14754 and the OneFlorida Clinical Research Consortium (CDRN-1501-26692). The content is solely the responsibility of the authors and does not necessarily represent the official views of the NIH or PCORI.

## AUTHOR CONTRIBUTIONS

JB, BH, and ES designed the initial concepts and framework for the proposed systematic scoping reviewing; TL, AL, and JB carried out the review and annotation process; TL, AL, and JB wrote the initial draft of the manuscript. TM, GL, YG, MP, YW, CH, TG, ES, and BH provided critical feedback and edited the manuscript.

## SUPPLEMENTARY MATERIAL

Supplementary material is available at *Journal of the American Medical Informatics Association* online.

## CONFLICT OF INTEREST STATEMENT

None declared.

## Supplementary Material

ocaa245_supplementary_data

## References

[1] Collins FS, Hudson KL, Briggs JP, Lauer MS. PCORnet: turning a dream into reality. J Am Med Inform Assoc 2014; 21 (4): 576–7. 24821744 10.1136/amiajnl-2014-002864 PMC4078299

[2] Corley DA, Feigelson HS, Lieu TA, McGlynn EA. Building data infrastructure to evaluate and improve quality: PCORnet. J Oncol Pract 2015; 11 (3): 204–6. 25980016 10.1200/JOP.2014.003194 PMC4438109

[3] PCORnet. Data-Driven | The National Patient-Centered Clinical Research Network. 2019. https://pcornet.org/data-driven-common-model/Accessed July 21, 2020.

[4] Shenkman E, Hurt M, Hogan W, et al OneFlorida Clinical Research Consortium: linking a clinical and translational science institute with a community-based distributive medical education model. Acad Med 2018; 93 (3): 451–5. 29045273 10.1097/ACM.0000000000002029 PMC5839715

[5] OneFlorida. OneFlorida Clinical Research Consortium. 2020. https://onefloridaconsortium.org/Accessed July 21, 2020

[6] US FDA. Real-World Evidence. 2020. https://www.fda.gov/science-research/science-and-research-special-topics/real-world-evidenceAccessed July 21, 2020

[7] Sherman RE, Anderson SA, Dal Pan GJ, et al Real-world evidence—what is it and what can it tell us? N Engl J Med 2016; 375 (23): 2293–7. 27959688 10.1056/NEJMsb1609216

[8] Olsen L, Aisner D, McGinnis JM, Institute of Medicine (U.S.), eds. The Learning Healthcare System: Workshop Summary. Washington, DC: The National Academies Press; 2007. 21452449

[9] Budrionis A, Bellika JG. The learning healthcare system: where are we now? A systematic review. J Biomed Inform 2016; 64: 87–92. 27693565 10.1016/j.jbi.2016.09.018

[10] Sox HC. Comparative effectiveness research: a report from the Institute of Medicine. Ann Intern Med 2009; 151 (3): 203. 19567618 10.7326/0003-4819-151-3-200908040-00125

[11] Ford I, Norrie J. Pragmatic trials. N Engl J Med 2016; 375 (5): 454–63. 27518663 10.1056/NEJMra1510059

[12] Botsis T, Hartvigsen G, Chen F, Weng C. Secondary use of EHR: data quality issues and informatics opportunities. Summit Transl Bioinform 2010; 2010: 1–5. PMC3041534 21347133

[13] Bae CJ, Griffith S, Fan Y, et al The challenges of data quality evaluation in a joint data warehouse. eGEMs 2015; 3 (1): 12. 10.13063/2327-9214.1125 PMC4537084 26290882

[14] Wang RY, Strong DM. Beyond accuracy: what data quality means to data consumers. J Manag Infn Systems 1996; 12 (4): 5–33.

[15] McGilvray D. Executing Data Quality Projects: Ten Steps to Quality Data and Trusted Information. *MIT Information Quality Industry Symposium* 2009. http://mitiq.mit.edu/IQIS/Documents/CDOIQS_200977/Papers/01_02_T1D.pdf Accessed July 21, 2020.

[16] Chan KS, Fowles JB, Weiner JP. Review: electronic health records and the reliability and validity of quality measures: a review of the literature. Med Care Res Rev 2010; 67 (5): 503–27. 20150441 10.1177/1077558709359007

[17] Nahm M. Data quality in clinical research In: Richesson RL, Andrews JE, eds. Clinical Research Informatics. London: Springer; 2012: 175–201.

[18] Kahn MG, Raebel MA, Glanz JM, Riedlinger K, Steiner JF. A pragmatic framework for single-site and multisite data quality assessment in electronic health record-based clinical research. Medl Care 2012; 50: S21–9. 10.1097/MLR.0b013e318257dd67 PMC3833692 22692254

[19] Weiskopf NG, Weng C. Methods and dimensions of electronic health record data quality assessment: enabling reuse for clinical research. J Am Med Inform Assoc 2013; 20 (1): 144–51. 22733976 10.1136/amiajnl-2011-000681 PMC3555312

[20] Weiskopf NG, Hripcsak G, Swaminathan S, Weng C. Defining and measuring completeness of electronic health records for secondary use. J Biomed Inform 2013; 46 (5): 830–6. 23820016 10.1016/j.jbi.2013.06.010 PMC3810243

[21] Liaw ST, Rahimi A, Ray P, et al Towards an ontology for data quality in integrated chronic disease management: a realist review of the literature. Intl J Med Inform 2013; 82 (1): 10–24. 10.1016/j.ijmedinf.2012.10.001 23122633

[22] Zozus MN, Hammond WE, Green BB, et al Assessing Data Quality for Healthcare Systems Data Used in Clinical Research (Version 1.0). https://dcricollab.dcri.duke.edu/sites/NIHKR/KR/Assessing-data-quality_V1%200.pdf#search=Assessing%20Data%20Quality%20for%20Healthcare%20Systems%20Data%20Used%20in%20Clinical%20Research Accessed July 21, 2020.

[23] Johnson SG, Speedie S, Simon G, Kumar VWestra BL. A Data Quality Ontology for the Secondary Use of EHR Data. *AMIA Annu Symp Proc* 2015; 2015: 1937–46. PMC4765682 26958293

[24] Garcí A-de-León-Chocano R, Sáez C, Muñoz-Soler V, Garcí A-de-León-González R, García-Gómez JM. Construction of quality-assured infant feeding process of care data repositories: definition and design (Part 1). Comput Biol Med 2015; 67: 95–103. 26513467 10.1016/j.compbiomed.2015.09.024

[25] Kahn MG, Callahan TJ, Barnard J, et al A harmonized data quality assessment terminology and framework for the secondary use of electronic health record data. eGEMs 2016; 4 (1): 18. 10.13063/2327-9214.1244 PMC5051581 27713905

[26] Reimer AP, Milinovich A, Madigan EA. Data quality assessment framework to assess electronic medical record data for use in research. Int J Med Inform 2016; 90: 40–7. 27103196 10.1016/j.ijmedinf.2016.03.006 PMC4845906

[27] Khare R, Utidjian L, Ruth BJ, et al A longitudinal analysis of data quality in a large pediatric data research network. J Am Med Inform Assoc 2017; 24 (6): 1072–9. 28398525 10.1093/jamia/ocx033 PMC6259665

[28] Smith M, Lix LM, Azimaee M, et al Assessing the quality of administrative data for research: a framework from the Manitoba Centre for Health Policy. J Am Med Inform Assoc 2018; 25 (3): 224–9. 29025002 10.1093/jamia/ocx078 PMC7651885

[29] Weiskopf NG, Bakken S, Hripcsak G, Weng C. A data quality assessment guideline for electronic health record data reuse. *EGEMS (Wash DC)* 2017; 5 (1): 14. 10.5334/egems.218 PMC5983018 29881734

[30] Lee K, Weiskopf N, Pathak J. A Framework for Data Quality Assessment in Clinical Research Datasets. *AMIA Annu Symp Proc* 2018; 2017: 1080–9. PMC5977591 29854176

[31] Feder SL. Data quality in electronic health records research: quality domains and assessment methods. West J Nurs Res 2018; 40 (5): 753–66. 28322657 10.1177/0193945916689084

[32] Terry AL, Stewart M, Cejic S, et al A basic model for assessing primary health care electronic medical record data quality. BMC Med Inform Decis Mak 2019; 19 (1): 30. 30755205 10.1186/s12911-019-0740-0 PMC6373085

[33] Nordo A, Eisenstein EL, Garza M, Hammond WE, Zozus MN Evaluative outcomes in direct extraction and use of EHR data in clinical trials. Stud Health Technol Inform 2019; 257: 333–40. 30741219

[34] Bloland P, MacNeil A. Defining & assessing the quality, usability, and utilization of immunization data. BMC Public Health 2019; 19 (1): 19. 30947703 10.1186/s12889-019-6709-1 PMC6450010

[35] Henley-Smith S, Boyle D, Gray K. Improving a secondary use health data warehouse: proposing a multi-level data quality framework. eGEMs 2019; 7 (1): 38. 31531384 10.5334/egems.298 PMC6676919

[36] Charnock V. Electronic healthcare records and data quality. Health Info Libr J 2019; 36 (1): 91–5. 30811882 10.1111/hir.12249

[37] Callahan TJ, Bauck AE, Bertoch D, et al A comparison of data quality assessment checks in six data sharing networks. eGEMs 2017; 5 (1): 8. 10.5334/egems.223 PMC5982846 29881733

[38] Qualls LG, Phillips TA, Hammill BG, et al Evaluating foundational data quality in the National Patient-Centered Clinical Research Network (PCORnet). eGEMs 2018; 6 (1): 3 29881761 10.5334/egems.199 PMC5983028

[39] PCORnet. PCORnet Data Checks, version 8. 2020. https://pcornet.org/wp-content/uploads/2020/03/PCORnet-Data-Checks-v8.pdfAccessed July 21

[40] Strong DM, Lee YW, Wang RY. Data quality in context. Commun ACM 1997; 40 (5): 103–10.

[41] Mocnik F-B, Mobasheri A, Griesbaum L, Eckle M, Jacobs C, Klonner C. A grounding-based ontology of data quality measures. JOSIS 2018; (16): 1–25.

[42] Kahn MG, Brown JS, Chun AT, et al Transparent reporting of data quality in distributed data networks. eGEMs 2015; 3 (1): 7. 10.13063/2327-9214.1052 PMC4434997 25992385

