# Supplementary material for: Assessing the practice of data quality evaluation in a national clinical data research network through a systematic scoping review in the era of real-world data
Source: J Am Med Inform Assoc. 2020 Nov 9;27(12):1999–2010. doi: 10.1093/jamia/ocaa245 (PMC7727392; doi:10.1093/jamia/ocaa245)
Supplement: ocaa245_supplementary_data [file ocaa245_supplementary_data.docx]

# **APPENDIX A. Search query for additional studies related to electronic health record data quality.**

| **Database** | **Search** | **Restrictions** |
| --- | --- | --- |
| PubMed | ("data quality"[Title/Abstract] OR "data accuracy"[Title/Abstract] OR "data reliability"[Title/Abstract] OR "data validity"[Title/Abstract] OR "data consistency"[Title/Abstract] OR "data completeness"[Title/Abstract] OR "data error"[Title/Abstract] OR "performance measure"[Title/Abstract] OR "quality measure"[Title/Abstract] OR "quality measurement"[Title/Abstract] OR "quality assurance"[Title/Abstract] OR "missing data"[Title/Abstract] OR "data use"[Title/Abstract] OR "data evaluation"[Title/Abstract] OR "data verification"[Title/Abstract]) AND ("EHR"[Title/Abstract] OR "electronic medical record"[Title/Abstract] OR "computerized medical record"[Title/Abstract] OR "computerized medical record system"[Title/Abstract] OR "electronic health record"[Title/Abstract] OR "electronic patient record"[Title/Abstract] OR "administrative data"[Title/Abstract] OR "computerized patient record"[Title/Abstract] OR "computerised medical record"[Title/Abstract] OR "EMR"[Title/Abstract] OR "EHRs"[Title/Abstract] OR "electronic medical records"[Title/Abstract] OR "computerized medical records"[Title/Abstract] OR "computerized medical record systems"[Title/Abstract] OR "electronic health records"[Title/Abstract] OR "electronic patient records"[Title/Abstract] OR "administrative datas"[Title/Abstract] OR "computerized patient records"[Title/Abstract] OR "computerised medical records"[Title/Abstract] OR "EMRs"[Title/Abstract]) | Published from 2012-Jan. to 2020-Feb.  English Language |

# **APPENDIX B.**

Included reviews and frameworks.

| **PMID** | **Title** |
| --- | --- |
| 30755205 | A basic model for assessing primary health care electronic medical record data quality. |
| 29854176 | A Framework for Data Quality Assessment in Clinical Research Datasets. |
| 28322657 | Data Quality in Electronic Health Records Research: Quality Domains and Assessment Methods |
| 28398525 | A longitudinal analysis of data quality in a large pediatric data research network. |
| 29025002 | Assessing the quality of administrative data for research: a framework from the Manitoba Centre for Health Policy. |
| 29881734 | A Data Quality Assessment Guideline for Electronic Health Record Data Reuse. |
| 27103196 | Data quality assessment framework to assess electronic medical record data for use in research. |
| 26513467 | Construction of quality-assured infant feeding process of care data repositories: definition and design (Part 1) |
| 30947703 | Defining & assessing the quality, usability, and utilization of immunization data |
| 31531384 | Improving a Secondary Use Health Data Warehouse: Proposing a Multi-Level Data Quality Framework |
| 30811882 | Electronic healthcare records and data quality. |

Included 139 individual data quality assessment studies.

| **PMID** | **Title** |
| --- | --- |
| 30616535 | Providing quality data in health care - almost perfect inter-rater agreement in the Norwegian tonsil surgery register. |
| 30741240 | Rule-Based Data Quality Assessment and Monitoring System in Healthcare Facilities. |
| 31438254 | Representing Rules for Clinical Data Quality Assessment Based on OpenEHR Guideline Definition Language. |
| 31819655 | Validation Of Cancer Diagnoses In Electronic Health Records: Results From The Information System For Research In Primary Care (SIDIAP) In Northeast Spain. |
| 28408759 | Reliably estimating prevalences of atopic children: an epidemiological study in an extensive and representative primary care database. |
| 31599645 | Noncurated Data Lead to Misinterpretation of Treatment Outcomes in Patients With Prostate Cancer After Salvage or Palliative Radiotherapy. |
| 31824196 | Developing a Registry of Healthcare-Associated Infections at Intensive Care Units in West China: Study Rationale and Patient Characteristics. |
| 31437950 | Detecting Systemic Data Quality Issues in Electronic Health Records. |
| 31408470 | Data quality self-assessment of child health and sexual reproductive health indicators in Botswana, 2016-2017. |
| 31349037 | Novel Data Linkages to Characterize Palliative and End-Of-Life Care: Challenges and Considerations. |
| 31785526 | Examining the impact of data quality and completeness of electronic health records on predictions of patients' risks of cardiovascular disease. |
| 30896435 | Completeness of Electronic Dental Records in a Student Clinic: Retrospective Analysis. |
| 31438009 | Differences Between What Is Said During the Consultation and What Is Recorded in the Electronic Health Record. |
| 23650483 | Developing a tool to assess the quality of socio-demographic data in community health centres. |
| 30488750 | The assessment of data quality issues for process mining in healthcare using Medical Information Mart for Intensive Care III, a freely available e-health record database. |
| 25590511 | Completeness and accuracy of the wisconsin immunization registry: an evaluation coinciding with the beginning of meaningful use. |
| 30487299 | Establishing a composite neonatal adverse outcome indicator using English hospital administrative data. |
| 31531382 | Design and Refinement of a Data Quality Assessment Workflow for a Large Pediatric Research Network. |
| 25554683 | Visual grids for managing data completeness in clinical research datasets. |
| 31438205 | Towards Structured Data Quality Assessment in the German Medical Informatics Initiative: Initial Approach in the MII Demonstrator Study. |
| 27653186 | The accuracy of claims data for measuring transfusion rates. |
| 31412826 | Use of electronic health records from a statewide health information exchange to support public health surveillance of diabetes and hypertension. |
| 31777787 | Differences in Longitudinal Disease Activity Between Research Cohort and Noncohort Participants with Rheumatoid Arthritis Using Electronic Health Record Data. |
| 24403981 | Validating emergency department vital signs using a data quality engine for data warehouse. |
| 31367649 | DataGauge: A Practical Process for Systematically Designing and Implementing Quality Assessments of Repurposed Clinical Data. |
| 30497872 | Initializing a hospital-wide data quality program. The AP-HP experience. |
| 31445249 | Strengths, pitfalls, and lessons learned in implementing electronic collection of childhood vaccination data in Zambia: The SmartCare experience. |
| 31945461 | Assessing Quality of Surgical Real-World Data from an Automated Electronic Health Record Pipeline. |
| 25220487 | Does single-source create an added value? Evaluating the impact of introducing x4T into the clinical routine on workflow modifications, data quality and cost-benefit. |
| 25884526 | Approach to addressing missing data for electronic medical records and pharmacy claims data research. |
| 30114277 | The United Kingdom National Neonatal Research Database: A validation study. |
| 31145441 | Medication Accuracy in Electronic Health Records for Microbial Keratitis. |
| 31425392 | Evaluating HIV Surveillance Completeness Along the Continuum of Care: Supplementing Surveillance With Health Center Data to Increase HIV Data to Care Efficiency. |
| 31061618 | Measuring the quality and completeness of medication-related information derived from hospital electronic health records database. |
| 31444047 | International Validation of the Danish Vascular Registry Karbase: A Vascunet Report. |
| 28687707 | Validity of Cardiovascular Data From Electronic Sources: The Multi-Ethnic Study of Atherosclerosis and HealthLNK. |
| 29726427 | Cleansing and Imputation of Body Mass Index Data and Its Impact on a Machine Learning Based Prediction Model. |
| 31365089 | Challenges with quality of race and ethnicity data in observational databases. |
| 29027512 | Challenges associated with missing data in electronic health records: A case study of a risk prediction model for diabetes using data from Slovenian primary care. |
| 23911177 | Challenges and future directions of the T1D Exchange Clinic Network and registry. |
| 29254419 | Accuracy of inter-hospital transfer information in Australian hospital administrative databases. |
| 31185983 | Accuracy and quality of immunization data in Iran: findings from data quality self-assessment survey in 2017. |
| 31842960 | A systematic review identifying common data items in neonatal trials and assessing their completeness in routinely recorded United Kingdom national neonatal data. |
| 31259018 | A Framework for Visualizing Data Quality for Predictive Models and Clinical Quality Measures. |
| 25347050 | Consistency of denominator data in electronic health records in Australian primary healthcare services: enhancing data quality. |
| 25169464 | Addressing electronic clinical information in the construction of quality measures. |
| 23823186 | Multi-Institutional Sharing of Electronic Health Record Data to Assess Childhood Obesity. |
| 26290882 | The Challenges of Data Quality Evaluation in a Joint Data Warehouse. |
| 28056955 | Development of an algorithm for determining smoking status and behaviour over the life course from UK electronic primary care records. |
| 24864178 | Variation in outcomes of quality measurement by data source. |
| 29728246 | Quality and accuracy of electronic pre-anesthesia evaluation forms. |
| 26590376 | Community-acquired pneumonia (CAP) hospitalizations and deaths: is there a role for quality improvement through inter-hospital comparisons? |
| 30349630 | A Comparison of Electronic Medical Record Data to Paper Records in Antiretroviral Therapy Clinic in Ethiopia: What is affecting the Quality of the Data? |
| 25203630 | Failure-to-rescue and interprovider comparisons after elective abdominal aortic aneurysm repair. |
| 27769288 | Comparing routine administrative data with registry data for assessing quality of hospital care in patients with myocardial infarction using deterministic record linkage. |
| 26133382 | Are administrative data valid when measuring patient safety in hospitals? A comparison of data collection methods using a chart review and administrative data. |
| 22337532 | Validity of eight integrated healthcare delivery organizations' administrative clinical data to capture breast cancer chemotherapy exposure. |
| 27107454 | Data quality of electronic medical records in Manitoba: do problem lists accurately reflect chronic disease billing diagnoses? |
| 28709453 | Validation of multisource electronic health record data: an application to blood transfusion data. |
| 30394981 | Variation in Laboratory Test Naming Conventions in EHRs Within and Between Hospitals: A Nationwide Longitudinal Study. |
| 24551421 | Sick patients have more data: the non-random completeness of electronic health records. |
| 29016972 | Biases introduced by filtering electronic health records for patients with "complete data". |
| 23707000 | Operational data integrity during electronic health record implementation in the ED. |
| 29771350 | Accuracy of the medication list in the electronic health record-implications for care, research, and improvement. |
| 24993545 | Using the CER Hub to ensure data quality in a multi-institution smoking cessation study. |
| 28221896 | Accuracy and Thoroughness of Treatment Summaries Provided as Part of Survivorship Care Plans Prepared by Two Cancer Centers. |
| 29027512 | Challenges associated with missing data in electronic health records: A case study of a risk prediction model for diabetes using data from Slovenian primary care. |
| 27013297 | The completeness of electronic medical record data for patients with Type 2 Diabetes in primary care and its implications for computer modelling of predicted clinical outcomes. |
| 25954416 | Concordance of Electronic Health Record (EHR) Data Describing Delirium at a VA Hospital. |
| 26262187 | Evaluating the data completeness in the Electronic Health Record after the Implementation of an Outpatient Electronic Health Record. |
| 28118921 | Reusability of coded data in the primary care electronic medical record: A dynamic cohort study concerning cancer diagnoses. |
| 29970009 | Assessment of the Feasibility of automated, real-time clinical decision support in the emergency department using electronic health record data. |
| 26460104 | Application of process mining to assess the data quality of routinely collected time-based performance data sourced from electronic health records by validating process conformance. |
| 28853764 | Exploring Vital Sign Data Quality in Electronic Health Records with Focus on Emergency Care Warning Scores. |
| 28500199 | Data quality in electronic medical records in Manitoba: Do problem lists reflect chronic disease as defined by prescriptions? |
| 27825333 | Data extraction from electronic health records (EHRs) for quality measurement of the physical therapy process: comparison between EHR data and survey data. |
| 24394554 | Real-time database drawn from an electronic health record for a thoracic surgery unit: high-quality clinical data saving time and human resources. |
| 25954462 | Improving Clinical Data Integrity by using Data Adjudication Techniques for Data Received through a Health Information Exchange (HIE). |
| 29881761 | Evaluating Foundational Data Quality in the National Patient-Centered Clinical Research Network (PCORnet¬Æ). |
| 26620698 | An assessment of data quality in a multi-site electronic medical record system in Haiti. |
| 28619704 | Applying STOPP Guidelines in Primary Care Through Electronic Medical Record Decision Support: Randomized Control Trial Highlighting the Importance of Data Quality. |
| 29854208 | Challenges with Collecting Smoking Status in Electronic Health Records. |
| 26951273 | Validation of Stroke Meaningful Use Measures in a National Electronic Health Record System. |
| 28154835 | Design of the New York City Macroscope: Innovations in Population Health Surveillance Using Electronic Health Records. |
| 30774449 | The internal validation of weight and weight change coding using weight measurement data within the UK primary care Electronic Health Record. |
| 29668691 | The impact of routine data quality assessments on electronic medical record data quality in Kenya. |
| 22150637 | Data integrity module for data quality assurance within an e-health system in sub-Saharan Africa. |
| 30398453 | Can we trust Electronic health records? The Smoking Test for Commission Errors. |
| 28154837 | Can Electronic Health Records Be Used for Population Health Surveillance? Validating Population Health Metrics Against Established Survey Data. |
| 25848737 | Reliability study of clinical electronic records with paper records in the NSW Public Oral Health Service. |
| 28765864 | Comparison of EHR-based diagnosis documentation locations to a gold standard for risk stratification in patients with multiple chronic conditions. |
| 29425633 | Electronic medical records as a replacement for prospective research data collection in postoperative pain and opioid response studies. |
| 26635485 | Validation of International Classification of Diseases coding for bone metastases in electronic health records using technology-enabled abstraction. |
| 25160168 | EHR-based disease registries to support integrated care in a health neighbourhood: an ontology-based methodology. |
| 22313561 | Health reform: is routinely collected electronic information fit for purpose? |
| 29512333 | Study design and baseline characteristics of inpatients with diabetes mellitus in a tertiary hospital in China: A database study based on electronic medical records. |
| 28350996 | Quality measures of the population-based Finnish Cancer Registry indicate sound data quality for solid malignant tumours. |
| 26661718 | Assessing race and ethnicity data quality across cancer registries and EMRs in two hospitals. |
| 29282723 | Comparison of Electronic Health Record-Based and Claims-Based Diabetes Care Quality Measures: Causes of Discrepancies. |
| 30488750 | The assessment of data quality issues for process mining in healthcare using Medical Information Mart for Intensive Care III, a freely available e-health record database. |
| 23514203 | Evaluation of data completeness in the electronic health record for the purpose of patient recruitment into clinical trials: a retrospective analysis of element presence. |
| 30487299 | Establishing a composite neonatal adverse outcome indicator using English hospital administrative data. |
| 27081408 | Application of An Ontology for Characterizing Data Quality For a Secondary Use of EHR Data. |
| 28357037 | Electronic medical records in humanitarian emergencies - the development of an Ebola clinical information and patient management system. |
| 21994091 | Multicentre study of the quality of a large administrative data set and implications for comparing death rates. |
| 30422782 | Assessing Brief Intervention for Unhealthy Alcohol Use: A Comparison of Electronic Health Record Documentation and Patient Self-Report. |
| 24378608 | Evaluation of immunization data completeness within a large community health care system exchanging data with a state immunization information system. |
| 25528664 | Sharing of clinical data in a maternity setting: how do paper hand-held records and electronic health records compare for completeness? |
| 26252212 | Implementation of a Cloud-Based Electronic Medical Record to Reduce Gaps in the HIV Treatment Continuum in Rural Kenya. |
| 25670229 | Implementation of a cloud-based electronic medical record for maternal and child health in rural Kenya. |
| 28210424 | Deployment of Analytics into the Healthcare Safety Net: Lessons Learned. |
| 29500200 | Linkage of Maternity Hospital Episode Statistics data to birth registration and notification records for births in England 2005-2014: Quality assurance of linkage of routine data for singleton and multiple births. |
| 27429992 | Using a Data Quality Framework to Clean Data Extracted from the Electronic Health Record: A Case Study. |
| 25316876 | Limitations of using administratively reported immunization data for monitoring routine immunization system performance in Nigeria. |
| 28542065 | Data for Community Health Assessment in Rural Colorado: A Comparison of Electronic Health Records to Public Health Surveys to Describe Childhood Obesity. |
| 22583552 | Measuring data reliability for preventive services in electronic medical records. |
| 27876636 | Concordance Between Veterans' Self-Report and Documentation of Surrogate Decision Makers: Implications for Quality Measurement. |
| 25954362 | Validating Health Information Exchange (HIE) Data For Quality Measurement Across Four Hospitals. |
| 24471935 | Using computer-extracted data from electronic health records to measure the quality of adolescent well-care. |
| 26950399 | Construction of quality-assured infant feeding process of care data repositories: Construction of the perinatal repository (Part 2). |
| 29069394 | Exploring completeness in clinical data research networks with DQe-c. |
| 25880660 | Using an electronic medical record (EMR) to conduct clinical trials: Salford Lung Study feasibility. |
| 29866625 | Benchmarking emergency department thoracotomy: Using trauma video review to generate procedural norms. |
| 24411310 | Parity derived for pregnant women using historical administrative hospital data: accuracy varied among patient groups. |
| 29253138 | Success factors for implementing and sustaining a mature electronic medical record in a low-resource setting: a case study of iSant√© in Haiti. |
| 24721489 | Influence of data quality on computed Dutch hospital quality indicators: a case study in colorectal cancer surgery. |
| 23920607 | Routinely-collected general practice data from the electronic patient record and general practitioner active electronic questioning method: a comparative study. |
| 28453637 | Automated identification of implausible values in growth data from pediatric electronic health records. |
| 25818915 | Small rural emergency services can electronically collect accurate episode-level data: A cross-sectional study. |
| 30497872 | Initializing a hospital-wide data quality program. The AP-HP experience. |
| 30815075 | Interoperability Progress and Remaining Data Quality Barriers of Certified Health Information Technologies. |
| 29756355 | Development and Validation of a High-Quality Composite Real-World Mortality Endpoint. |
| 29717951 | Completeness and accuracy of anthropometric measurements in electronic medical records for children attending primary care. |
| 23453115 | Data quality of an electronic health record tool to support VA cardiac catheterization laboratory quality improvement: the VA Clinical Assessment, Reporting, and Tracking System for Cath Labs (CART) program. |
| 28701374 | Hospital admission rates and emergency department use in relation to glycated hemoglobin in people with diabetes mellitus: a linkage study using electronic medical record and administrative data in Ontario. |
| 29475824 | Characterizing and Managing Missing Structured Data in Electronic Health Records: Data Analysis. |
| 28912200 | Development and Validation of Electronic Quality Measures to Assess Care for Patients With Transient Ischemic Attack and Minor Ischemic Stroke. |
| 22737097 | Identifying patients with hypertension: a case for auditing electronic health record data. |
| 24145818 | Is the quality of data in an electronic medical record sufficient for assessing the quality of primary care? |
